# Improving the Performance of Catalytic Combustion Type Methane Gas Sensors Using Nanostructure Elements Doped with Rare Earth Cocatalysts

**DOI:** 10.3390/s110100019

**Published:** 2010-12-23

**Authors:** Ying Wang, Min Ming Tong, Dan Zhang, Zhen Gao

**Affiliations:** 1 Research Institution of Methane and Safety Monitoring Technology, China University of Mining and Technology, China; E-Mail: jctmm@163.com; 2 Faculty of Engineering and Applied Science, University of Ontario Institute of Technology, Oshawa, ON L1H 7K4, Canada; E-Mails: Dan.Zhang@uoit.ca (D.Z); Zhen.Gao@uoit.ca (Z.G.)

**Keywords:** methane gas sensor, catalytic combustion, cerium, nanostructure elements

## Abstract

Conventional methane gas sensors based on catalytic combustion have the drawbacks of high working temperature, low thermal stability and small measurement range. To improve their performance, cerium, which possesses high oxygen storage and release ability, was introduced via nanotechnology to prepare Ce-contained nanostructure elements. Three kinds of elements with different carriers: Al_2_O_3_, n-Al_2_O_3_ and n-Ce-Al_2_O_3_ were prepared and separately fabricated (Pt-Pd/Al, Pt-Pd/n-Al, Pt-Pd/n-Ce-Al). The performances of Wheatstone Bridges with three different catalytic elements were tested and compared. The results indicated that the cerium-containing element exhibited better performance than other elements regarding activity, anti-sulfur ability and thermal stability. Moreover, a constant temperature circuit was also applied in this system. The measurement range was extended from 4% to 10% by automatically decreasing the working current in a reasonable range. The maximum error for 0%–10% CH_4_ was controlled below 5%, which fully meets the measurement requirements.

## 1. Introduction

Methane explosions are always one of the major risks in coal mining. In recent years, numerous methane explosion disasters have occurred in coal mines which have caused great human life and property losses, such as the mine explosion in West Virginia, USA in April 2010, in which 25 coal miners were killed or the mine explosion in ShanXi, China, where 150 coal miners lost their lives in the disaster. Similar disasters have also happened in Russia, *etc.* To decrease the risk of methane explosion, different types of methane sensors based on gas chromatography, semiconductor, infrared and optical interference *etc.* are developed to monitor and forewarn of the methane concentration [1,2]. However, stable performance of these detectors often cannot be achieved because of the limitations of the hostile coal mine environment. This is also one important reason why methane explosions occur so frequently.

**Figure 1.**
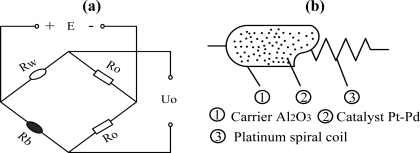
Methane sensors based on catalytic combustion. **(a)** Wheatstone bridge with catalytic element, **(b)** black element with carrier catalyst.

With a simple structure, low cost, stable performance in severe atmospheres, gas sensors based on catalytic combustion are used widely. Normally, methane concentration is detected via a Wheatstone bridge [as shown in Figure 1(a)], which is made up of a white element (Rw), a black element (Rb) (Rw = Rb) and two compensating resistances Ro. Conventionally, the white element is obtained by coating Al_2_O_3_ as carrier on a platinum wire. The black element is fabricated by depositing Pt, Pd or other transition metal oxides on the surface of the white element [as shown in Figure 1(b)].

The catalytic element is electrically heated to about 400 °C, at which temperature catalytic oxidation of the methane readily occurs. The rise in temperature, with the corresponding increase in the resistance of black element produced by reaction on the catalyst, is then measured by incorporating the element in the Wheatstone bridge network where the potential difference across the bridge forms the output of the device, as shown below:

(1) $${\mathrm{CH}}_{4}+O_{2}\overset{Pt-Pd}{\to }2H_{2}O+{\mathrm{CO}}_{2}+795.5kJ$$

The resistance of the black element increases to Rb + ΔRb, but the rest resistances in the bridge remains the same, thus the bridge becomes unbalanced and output voltage can be described as:

(2) $$U_{0}=\frac{R_{b}+\Delta R_{b}}{R_{w}+(R_{b}+\Delta R_{b})}E-\frac{E}{2}$$

Suppose Rw = Rb and far more than ΔRb, the expression can be changed to:

(3) $$U_{o}=\frac{E}{R_{w}+R_{b}}\Delta R_{b}=\frac{E}{2R}\Delta R_{b}=K\cdot \Delta R_{b}\;\;\;\;\;\;,(K=\frac{E}{2R})$$

In this way, methane concentration can be detected by measuring the variation of output voltage from this unbalanced Wheatstone bridge [3,4]. However, there are also some defects which limit the application: sintering of carrier Al_2_O_3_ and noble metal catalysts at continuous high temperature; sulfur poisoning of the catalytic element caused by the sulfur-containing atmosphere in the coal mine. As a result, thermal stability and activity of the catalytic elements decline. In addition, methane concentration is proportional to the working temperature, which must be controlled below 600 °C, and just 0–4% CH_4_ can be measured, which is too small for practical methane measurements.

In recent years, with the in-depth research on catalytic materials, people have paid more attention to the improvement of some special cocatalysts such as CeO_2_, La_2_O_3_, and ZrO_2_ in catalytic combustion. The catalytic performance can be enhanced significantly by doping these cocatalysts with pure carrier Al_2_O_3_ [5–7]. Some researchers have also focused on the use of nano techniques in the preparation of carriers and cocatalysts. Successful results have been obtained [8–10]. Tong’s team also investigated the performance of a catalytic methane sensor with nanostructure carrier alumina and perfect performance was also achieved [11]. However, only the modification of material is studied, and few investigations about the modification of methane catalytic elements (black element) in methane sensors have been reported.

Some researchers have also focused on the range extension of methane sensors, since the measurement range for conventional methane sensor based on catalytic combustion is only 0–4%, otherwise it will become an explosive source caused by the high temperature generated from catalytic combustion. Researchers at the Wuhan University of Technology tried to integrate catalytic combustion type methane sensors and heat conduction type methane sensors together, and 0–100% CH_4_ detection was achieved thereby, but the structure complexity and errors increased greatly [12]. Tong’s team also tries to use a constant temperature circuit in this system to extend the range in a previous study, and the detection range was extended considerably [13,14].

In this paper, we have developed a new nanostructure catalytic detection system with excellent performance with regards to activity, anti-sulfur ability and stability. The main idea was to improve the performance of methane sensors by modifying the black elements with a nanostructure cerium-containing material. Two modified catalytic elements: Pt-Pd/n-Al_2_O_3_ and Pt-Pd/n-Ce-Al_2_O_3_ and a conventional Pt-Pd/Al_2_O_3_ catalytic element were fabricated and compared in this work. Moreover, a constant temperature method was also applied to extend the measurement range and control the temperature during operation.

## 2. Experimental Section

### 2.1. Raw Materials

1. Aluminium nitrate [Al(NO_3_)_3_], AR, XuZhou MOL Medical Reagent Factory;
2. Cerous nitrate [Ce(NO_3_)_3_•6H_2_O], AR, XuZhou Industry and Trade Company;
3. Nitric acid (HNO_3_), AR, XuZhou MOL Medical Reagent Factory;
4. Ammonia (NH_3_•H_2_O), AR, XuZhou Pure Chemical Factory;
5. Absolute ethanol (CH_3_CH_2_OH), AR, XuZhou Pure Chemical Factory;
6. Palladium chloride solution (PdCl_2_), AR, self-prepared;
7. Chloroplatinic acid solution (H_2_PtCl_6_•6H_2_O), AR, self-prepared.

### 2.2. Preparation of Carriers

Three kinds of carriers: Al_2_O_3_, n-Al_2_O_3_ and n-Ce-Al_2_O_3_ were prepared in the experiments. The sol-gel method was adopted since it yields products with high purity, homogeneity, and well-controlled properties. Al(NO_3_)_3_, Ce(NO_3_)_3_•6H_2_O solution were mixed with a certain ratio (Ce:Al = 0.15:0.85). The mixed solution obtained was vigorously stirred before ammonia was added dropwise till pH > 9. The sample was then stirred for 3 more h and aged at room temperature before being filtered and washed with deionized water, and then supercritically dried in an oven overnight with ethanol as medium at 110 °C. In order to stabilize the performance, gel need to be calcined. n-Ce-Al_2_O_3_ was also prepared this way. Finally the n-Al_2_O_3_ material was also prepared similarly. The normal Al_2_O_3_ vector was obtained from common high-purity alumina. BET surface area was measured separately after all the samples were calcined at 500 and 1,000 °C.

### 2.3. Preparation of Catalytic Elements

**Figure 2.**
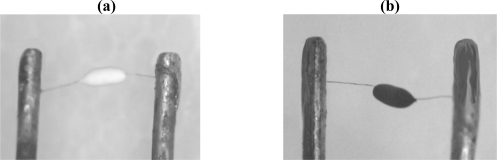
Catalytic elements before and after coating **(a)** Before coating, **(b)** After coating.

Three prepared carriers: Al_2_O_3_, n-Al_2_O_3_ and n-Ce-Al_2_O_3_ were mixed with adhesive and coated on a platinum coil separately, and then calcined in a muffle furnace according to a certain temperature curve. Catalytic elements before and after coating are shown in Figures 2(a,b). PdCl_2_ and H_2_PtCl_6_•6H_2_O solutions were allowed to absorb on the surface of carriers at the same time via an sometric impregnation method. Elements were dried and aged with 10% high concentration CH_4_ for 15 min, repeated three times, and spot welded. Thus, three black elements: Pt-Pd/Al_2_O_3_, Pt-Pd/n-Al_2_O_3_, Pt-Pd/n-Ce-Al_2_O_3_ were prepared.

The prepared white and black elements were matched by a dynamic matching method. The best matched elements were encapsulated with epoxy resin and connected as an electrical bridge. Bridge_Al_ stands for bridge containing Pt-Pd/Al_2_O_3_ catalytic element, bridge_n-Al_ stands for three bridge containing Pt-Pd/ n-Al_2_O_3_ catalytic element, and bridge_n-Ce-Al_ stands for bridge containing Pt-Pd/n-Ce-Al_2_O_3_ catalytic element.

### 2.4. Experiments to Examine Activity, Anti-Sulfur Ability and Stability

**Figure 3.**
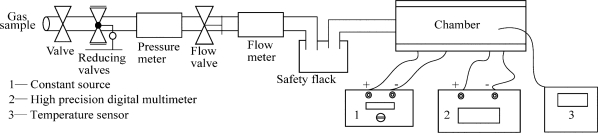
Experiment system for activity and thermal stability.

Briefly, Gas samples are introduced from the tubing, different electrical bridges are placed in the chamber, 3.0 V constant voltage from a constant voltage resource is applied to the electrical bridge, so that the errors caused by fluctuation and attenuation can be limited. The output of the electrical bridge is read by an AOIP high precision digital multimeter, while the temperature is detected by a temperature sensor (shown in Figure 3).

#### (1) Activity experiment:

This experiment was carried out to compare the reaction/working temperature of methane sensors with different catalytic elements. The bridges were placed in the chamber first before pre-heating for 10 minutes. The zero point was well adjusted at the work point, and then the bridges cooled down for testing. 1%–5% CH_4_ were introduced separately. Temperatures of catalytic elements were measured after the output of electrical bridges stablized.

#### (2) Anti-sulfur ability experiment:

This experiment was carried out to compare the anti-sulfur ability before and after sulfurated hydrogen was introduced. Three different electrical bridges were placed in the chamber. 1% CH_4_ was added first. After the output of the electrical bridge was stable, 120 ppm H_2_S gas was introduced. H_2_S gas was not allowed to stop until the catalytic elements were fully poisoned and the output was stable again. In the meantime, the outputs were recorded during the whole experiment. The zero points of the electrical bridges need to be adjusted before the experiment.

#### (3) Thermal stability experiment:

This experiment was carried out to compare the stability performance of sensors with different catalytic elements. Zero points of the six bridges were adjusted in pure air again before 1%, 2% pure CH_4_ gas was piped in the chamber separately. The output voltage was measured and recorded every 24 h.

## 3. Results and Discussion

### 3.1. Comparison of BET Surface Area

BET surface area and pore structure of the samples were observed using a Coulter Omnisorp-100CX system and high purity nitrogen as adsorption gas. BET surface area was calculated from the adsorptive data. Normally, pure alumina is not fully stable at the high temperatures that can be attained in both calcination and methane combustion, as it undergoes a phase change from *γ*-alumina to *α*-alumina, resulting in a loss of surface area and activity [15,16]. As shown in Table 1, the BET surface area of n-Al_2_O_3_ and n-Ce-Al_2_O_3_ are more than 220 m^2^/g after calcining at 500 °C. But after 1,000 °C calcinations, the surface areas of pure Al_2_O_3_ and n-Al_2_O_3_ are decreased drastically.

This problem can be partly solved by adding cerium as carrier to the pure alumina. As a unique cocatalyst with excellent oxygen storage and release ability, cerium can interact with Al_2_O_3_ to form a new ‘CeAlO_3_-precursor’ [17]. It can enhance the mechanical strength of Al_2_O_3_ and stabilize the surface area of Al_2_O_3_ by restraining the *γ*-Al_2_O_3_ phase change [18,19]. This is also proved in Table 1. The carrier that contains cerium still retains the much larger surface area than other carriers after calcining at 1,000 °C. Since catalysts with the larger BET surface area possess more active centers and larger reaction surface, better activity and sensitivity performance are achieved in cerium-contained elements.

### 3.2. Comparison of Activity

Reaction/working temperatures of six catalytic elements from the catalytic combustion in different CH_4_ atmospheres are summarized in Table 2. The reaction temperature sequence of these six bridges is: Bridge_n-Ce-Al_ < Bridge_n-Al_ < Bridge_Al_. Since carriers with a lower reaction temperature have a higher reaction activity, the doping of nanostructure carrier with rare earth cerium which has strong redox ability can enhance the activity performance.

This result is accordance with BET surface area result. More active centers contained in larger BET surface will enhance the activity performance of catalytic elements. Moreover, the range of measurement can be extended due to the decrease of reaction temperature.

### 3.3. Comparison of Anti-Sulfur Ability

**Figure 4.**
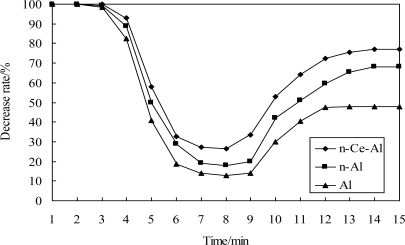
Anti-sulfur performance for three bridges.

The anti-sulfur ability of catalytic elements with different carriers is depicted in Figure 4, indicating that the sensitivity of all three electrical bridges was severely decreased and they were almost insensitive to methane while H_2_S was introduced. The sensitivity still can’t recover to the original conditions even if the H_2_S was removed later, and the output of the element containing Pt-Pd/Al was less than 50% after being poisoned by hydrogen sulfide gas. In contrast, the cerium-containing element still had a higher output than the other carriers whether H_2_S was either added or removed later, and the output after being poisoned was still more than 70%. The comparison implies that the cerium doping can enhance the anti-sulfur ability of methane sensors remarkably.

**Figure 5.**
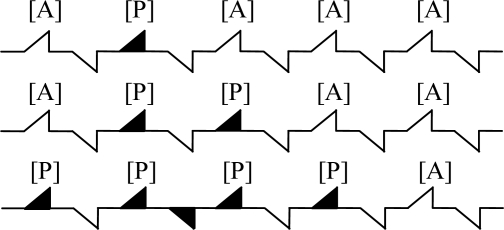
Theory of deactivation.

As the deactivation principle demonstrated in Figure 5 (note that A and P represents methane gas and the toxins from the reaction of Pt, Pd with H_2_S, SO_2_, NO_X_, respectively), the toxins are irreversibly absorbed by the active sites, and the number of active sites needed in the reaction is reduced with a subsequent poisoning of the catalyst. Through the continuous reaction process, the surface of catalyst was poisoned completely [20,21].

According to the previous effort [22], we conclude that the conductivity of oxide ions is of great importance for obtaining greater anti-sulfur ability. Because of the strong redox performance on the lattice surface, a Pt-Pd-CeO_2_ bond structure will form if CeO_2_ reacts with the Pt-Pd catalyst. Hence, this strong reaction leads to a large electron deficiency of the metal particles, which impedes the reaction between metal catalyst and sulfurous gas, and the active centers in catalyst can thereby be stabilized.

### 3.4. Comparison of Thermal Stability

**Figure 6.**
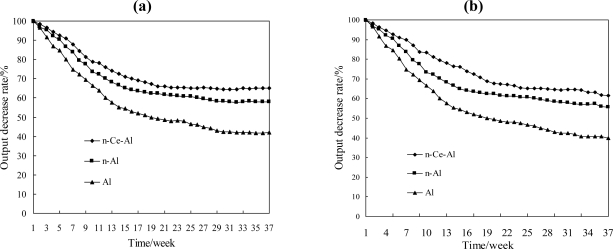
The stabilization comparison of three unit electrical bridges **(a)** 1% CH_4_, **(b)** 2% CH_4_.

A comparison of the thermal stability among Bridge_Al_, Bridge_n-Al_ and Bridge_n-Ce-Al_ is demonstrated in Figure 6. In 1% CH_4_ atmosphere, the output of the conventional methane elements Pt-Pd/Al_2_O_3_ and Pt-Pd/n-Al_2_O_3_ decreased by 58% and 42% within 10 months, while that of the element with the cerium decreased by only 35% [as shown in Figure 6(a)]. A similar result was also obtained in a 2% CH_4_ atmosphere experiment [as shown in Figure 6(b)]. The catalytic elements with CeO_2_ are much more stable. The SEM-EXD method was also applied to analyze the surface distribution of carriers. The results showed a very non-uniform Pt-Pd distribution over the surface of Pt-Pd/Al_2_O_3_, and while the concentration of Pt-Pd on part of the surface was quite high, elsewhere Pt-Pd could hardly be detected. In contrast, the distribution of Pt-Pd on the surface of Pt-Pd/n-Ce-Al_2_O_3_ exhibited better dispersion. Fullerton *et al*. confirmed that the decrease of the activity coefficient of Ce-γ-Al_2_O_3_ is less than that of pure γ-Al_2_O_3_ in a cerium-containing atmosphere, which is consistent with our experimental results [23].

It has been shown that, by doping cerium with pure alumina, the vacancies in alumina are easy to fill by Ce^3+^ ions, and the reversible oxidation reaction Ce_2_O_3_+1/2O_2_—2CeO_2_ occurs. The spinel structure possesses a strong ability to restrain the calcination. Hence, the output stability in the experiment is improved. From the above experiments on activity, anti-sulfur ability and thermal stability we can conclude that nanostructure Ce-containing catalytic elements exhibits much better performance than conventional catalytic elements used in methane sensors.

## 4. Detecting and Signal Processing System

**Figure 7.**
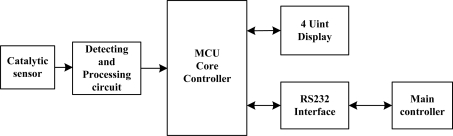
Methane detection system.

**Figure 8.**
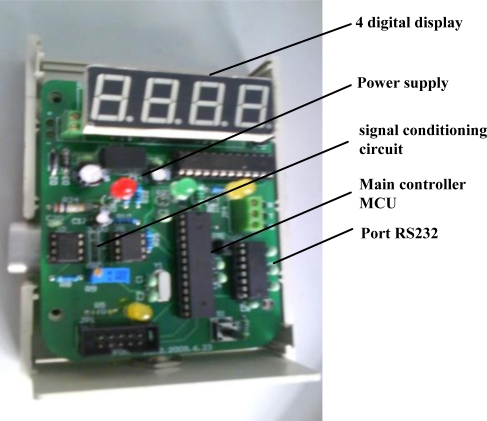
Signal processing module of the methane sensor.

The whole detection system can be described by Figure 7. Methane gas is first detected by the modified catalytic/black element in the methane sensor. The output from the unbalanced Wheatstone bridge, which is proportional to the concentration of methane is acquired and processed via the signal processing circuit and send it to the MCU for display and control. In this system, a single chip Atmega8 (Atmel company) is used as MCU in the detection module. A constant temperature circuit is adopted since it can extend the methane detection range and control the working temperature of the methane sensors. Real-time methane concentration is displayed by 4 unit digital displays (Figure 8) and also transferred to the main controller via a RS 232 serial port.

**Figure 9.**
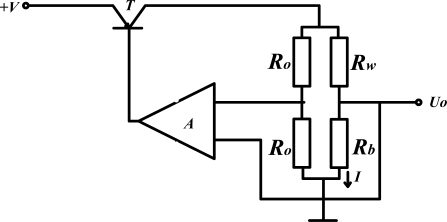
Measurement circuit with constant voltage.

The constant temperature circuit is simply presented in Figure 9. By adjusting the current I automatically, the working temperature as well as the resistance of catalytic element R_b_ remain constant. The methane gas concentration can be detected by measuring the current variation *ΔI* which is proportional to methane gas concentration [24,25]. In this way, the range of measurement is extended to 0–10%, and response time is shortened.

1% CH_4_ was used to calibrate the output of the whole system. However, the output signal and concentration of methane from the experiments exhibited nonlinear behavior, resulting in great errors during measurement. In order to eliminate the output errors, linear optimization needs to be introduced to ensure the output accuracy.

Firstly, the output signal mathematical model was set up. Static equation of heat balance for catalytic elements is described as follows:

(4) $$I^{2}R_{b}+\mu Y=\alpha S(T-T_{0})+A\sigma S(T^{4}-T_{0}^{4})$$

The left side of the equation represents the sum of the heat generated from the flow of the work current I across the catalytic element per unit time and the oxidative reaction of the inflammable gas over the catalytic element. The right-hand side of the equation is the sum of heat conduction and thermal radiation per unit time. When thermal equilibrium of the catalytic element is achieved, both sides of the equation are equivalent. T represents the temperature of the catalytic element, T_0_ represents the ambient temperature, R_b_ is the resistance value of the catalytic element, α, S, A, σ, μ represent physical parameters related to the materials and structure.

In the constant temperature methane detecting method, working current I decreases with the rise of gas concentration Y since the temperature of the catalytic element in a temperature constant state, T, and R_b_ are unchanged. In the condition of constant ambient temperature, the left side is constant. In this way, the equation can be expressed as:

(5) $$C=\alpha S(T-T_{0})+A\sigma S(T^{4}-T_{0}^{4}),\;\;\;\;\;C=I^{2}R_{b}+\mu Y$$

We assume the initial current is I_0_, *i.e.*, the working current in ambient normal atmospheric condition. C = I_0_^2^R_b_, equation (4) can be expressed as:

(6) $$I^{2}R_{b}+\mu Y=I_{0}{}^{2}R_{b}\;\;\;\;\mathrm{or}\;\;\;\;\;Y=(I_{0}{}^{2}R_{b}-I^{2}R_{b})/\mu$$

Suppose I = I_0_ − ΔI, then:

(7) $$\Delta I=I_{0}-\sqrt{I_{0}{}^{2}-\frac{\mu Y}{R_{b}}}$$

**Figure 10.**
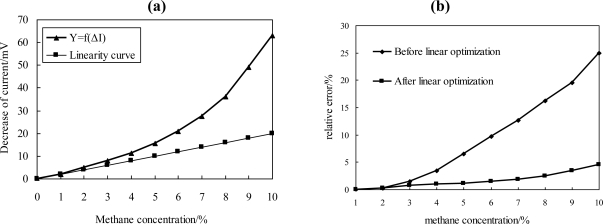
Linear optimization of the system. **(a)** Comparison of nonlinear output and linear curve. **(b)** Relative error before and after optimization.

As shown in Figure 10(a), the curve of ΔI = f(Y) is nonlinear. Thereby, the error between measurement value and true value becomes larger as the methane concentration increases. In this experiment, the measurement error for 0–4% CH_4_ is acceptable, but for concentrations above 5% CH_4_, the error is too large to be acceptable.

In order to extend the measurement range and eliminate this measurement error, linear optimization was applied to reduce the measurement error. In this experiment, a simple squares module was utilized, thus the correlation between current I^2^ and concentration of methane Y became linear. The relative error before and after linear optimization is presented in Figure 10(b); after optimization, the relative error declined significantly compared with the curve before linear optimization. The maximal error for detecting system in 0–10% CH_4_ atmosphere was below 5%, which already fully met the measurement requirements and national standards.

## 5. Conclusions

A methane gas detecting system was designed and tested in the paper. In order to enhance the performance, the rare earth cerium was applied to fabricate Ce-containing nanostructure elements in methane sensors. The experiment indicated that catalytic elements fabricated with cerium modified alumina have stronger oxygen storage and release ability than other conventional catalytic elements. It is easy for cerium to integrate with Al_2_O_3_ to form a ‘CeAlO_3_ precursor’, which restrains the change from *γ*-alumina to α-alumina, and also supplies enough O^2−^ in redox reactions, resulting in significant improvement in activity, anti-sulfur ability and thermal stability. Moreover, the constant temperature circuit was also introduced to extend the measurement range. By measuring the current variation caused by temperature increase, the methane concentration was obtained indirectly. In this way, the maximum measurement range was extended from 4% to 10% CH_4_. Additionally, linear optimization was utilized to eliminate the output error, which could be kept below 5% in the 0–10% CH_4_ range.

## Figures and Tables

**Table 1.** BET for three different carriers.

**BET**	**500 °C calcination/(m^2^/g)**	**1,000 °C calcination/(m^2^/g)**
Al_2_O_3_	98.2	43.7
n-Al_2_O_3_	234.4	72.3
n-Ce-Al_2_O_3_	223.7	91.7

**Table 2.** Reaction temperatures for different concentrations of methane.

**Temperature/°C**	**1%**	**2%**	**3%**	**4%**	**5%**
Bridge_Al1_	447.8	490.2	531.4	560.0	--
Bridge_Al2_	449.3	487.0	527.4	554.5	--
Bridge_n-Al1_	433.3	477.4	510.7	545.7	--
Bridge_n-Al2_	438.4	478.6	518.9	551.6	--
Bridge_n-Ce-Al1_	407.8	450.2	491.4	523.0	549.2
Bridge_n-Ce-Al2_	399.3	443.0	477.4	516.5	542.8
